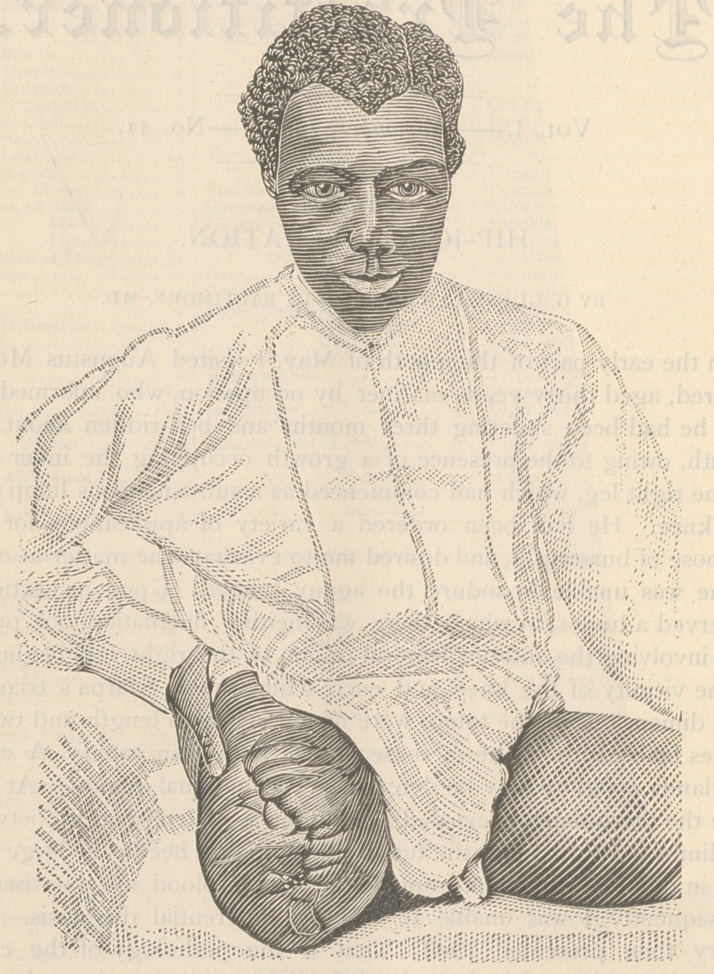# Hip-Joint Operation

**Published:** 1880-11

**Authors:** G. Glanville Rusk

**Affiliations:** Baltimore, Md.


### The Practitioner.

Vol. I.——*November, 1880.*——No. 11.

ARTICLE I.

### HIP-JOINT OPERATION.

BY G. GLANVILLE RUSK, M. D., BALTIMORE, MD.

In the early part of the month of May, I visited Augustus Moore, colored, aged thirty years, mariner by occupation, who informed me that he had been suffering three months and bed-ridden about one month, owing to the presence of a growth occupying the inner side of the right leg, which had commenced as a sub-cutaneous lump near the knee. He had been ordered a variety of applications for the purpose of bursting it, and desired me to evacuate the matter at once, as he was unable to indure the agony longer. Upon inspection I observed a tumor, in which there was neither fluctuation nor pulsation involving the anterior femoral region of the right leg, beginning in the vicinity of the knee, and encroaching upon Scarpa's triangle. The dimensions of the tumor were fifteen inches in length and twelve inches in breadth. The increase in size had been rapid. A chain of glands could readily be detected in the inguinal region. At this time the thermometer revealed no difference of temperature between the limbs. I thrust into the tumor an aspirating needle of large calibre, in different direction, from which neither blood nor pus issued; consequently, I was unable to make a differential diagnosis. The query then presented itself: what is the pathology of the case? From the evidence I had obtained, I was compelled to denominate it a soft sarcoma as the non-appearing of blood or pus contra-indicated the presence of an encysted abscess or an aneurism. My opinion was concurred in by several eminent professional brethren. In view of the man's deplorable condition, I proffered him an amputation at the hip-joint as the only source of hope for relief and recovery. He accepted gladly the proposition, notwithstanding I endeavored to impress upon his mind the great dangers attending such a procedure.

I sent him at once to the Church Home Infirmary for preparatory treatment as his vitality was at a low ebb. May 24th, at 2 P. M., I had him placed under the influence of chloroform for the purpose of making an extensive incision through the tumor, to verify or set aside my diagnosis, prior to operating. The contents of the tumor was coagulum. The circulation in the diseased limb had nearly ceased, lowering its temperature and gangrene seemed imminent; therefore, I proceeded to perform the amputation at the hip-joint, after the manner of Erichsen. Having used Lister's compressor the amount of hemorrhage was reduced to a minimum. Upon the removal of the limb, I dissected from the flaps some heterologous tissue, which unfortunately was not subjected to the miscroscope. After the ligation of the blood vessels, the edges of the wound were brought together and secured by silk sutures. I found the Nervis needle more convenient than the ordinary surgeon's needles. Re-action from surgical shock occurred in due time under the careful use of stimulants. No secondary hemorrhage. At my leisure I examined the tumor, which proved to be a diffused aneurism of the femoral artery.

May 25th. Rested well during the previous night under the influence of an anodyne, pulse 134, temperature 100.5.

May 26th. Pulse 120, temperature 101.2.

May 27th. Pulse 106, temperature 99.2.

May 28th. Pulse 110, temperature 98.6.

May 29th. Pulse 120, temperature 101.6.

May 30th. Pulse 128, temperature 101.8.

From the last mentioned date, till his dismissal from the Infirmary, his improvement was continuous. The treatment was an anodyne of opium or belladonna every night to secure rest; ten drops of the tincture of digitalis to calm the tumultuous action of his heart, alternated with quinine and iron every two hours as a tonic, and an enema of soap suds when required. The diet was meat essence, bread, milk and soft boiled eggs; no stimulants were used after re-action occurred. The stump, as well as the patient, was kept scrupulously clean; at no time was there any unpleasant odor from the wound.

June 14th. I discharged him from the infirmary well. Since that time he has engaged in the fruit packing business and is enjoying excellent health.

BALTIMORE, Oct. 7th, 1880.

## Figures and Tables

**Figure f1:**